# Regulation of P-glycoprotein and Breast Cancer Resistance Protein Expression Induced by Focused Ultrasound-Mediated Blood-Brain Barrier Disruption: A Pilot Study

**DOI:** 10.3390/ijms232415488

**Published:** 2022-12-07

**Authors:** Allegra Conti, Francoise Geffroy, Hermes A. S. Kamimura, Anthony Novell, Nicolas Tournier, Sébastien Mériaux, Benoit Larrat

**Affiliations:** 1 Université Paris-Saclay, CEA, CNRS, BAOBAB, NeuroSpin, 91191 Gif-sur-Yvette, France; 2 Department of Biomedicine and Prevention, University of Rome Tor Vergata, 00133 Rome, Italy; 3 Université Paris-Saclay, CEA, CNRS, Inserm, BioMaps, SHFJ, 91401 Orsay, France

**Keywords:** focused ultrasound, BBB-opening, efflux transporter, MRgFUS, drug delivery

## Abstract

The blood-brain barrier (BBB) controls brain homeostasis; it is formed by vascular endothelial cells that are physically connected by tight junctions (TJs). The BBB expresses efflux transporters such as P-glycoprotein (P-gp) and breast cancer resistance protein (BCRP), which limit the passage of substrate molecules from blood circulation to the brain. Focused ultrasound (FUS) with microbubbles can create a local and reversible detachment of the TJs. However, very little is known about the effect of FUS on the expression of efflux transporters. We investigated the in vivo effects of moderate acoustic pressures on both P-gp and BCRP expression for up to two weeks after sonication. Magnetic resonance-guided FUS was applied in the striatum of 12 rats. P-gp and BCRP expression were determined by immunohistochemistry at 1, 3, 7, and 14 days postFUS. Our results indicate that FUS-induced BBB opening is capable of (i) decreasing P-gp expression up to 3 days after sonication in both the treated and in the contralateral brain regions and is capable of (ii) overexpressing BCRP up to 7 days after FUS in the sonicated regions only. Our findings may help improve FUS-aided drug delivery strategies by considering both the mechanical effect on the TJs and the regulation of P-gp and BCRP.

## 1. Introduction

The blood-brain barrier (BBB) prevents macromolecules, small organic drugs, and ions from entering the brain, and it constitutes a major limit to central nervous system (CNS) pharmacotherapy [1,2]. The BBB comprises mainly endothelial cells connected by tight junctions (TJs) that, together with efflux transporters, can restrict the passage of substrate molecules from blood circulation to the brain [3,4,5,6]. ATP-binding cassette (ABC) transporters, such as the P-glycoprotein (P-gp) and breast cancer resistance protein (BCRP), are expressed at the BBB. Both P-gp and BRCP further minimize the transport of a large number of drugs to the brain, as observed with antiepileptic and anticancer drugs [5,7]. The P-gp and BCRP work in synergy, so the brain uptake of dual P-gp/BCRP substrates only increases when both P-gp and BCRP are down-regulated simultaneously. Indeed, if only one of the two pumps is down-regulated, then compensation by the other may occur, which still limits the molecular transport from the blood to the brain [8]. For example, it has been demonstrated that administering P-gp inhibitors can lead to an overall expression of BCRP [9].

Various approaches have been proposed to overcome BBB-related drug delivery challenges [10]. Among them, low-intensity focused ultrasound (FUS, typically referred to as intensities in the order of 1 W/cm^2^ in situ, compatible with acoustic pressures below 1.5 MPa) combined with circulating microbubbles (MBs) is a noninvasive technique that offers superior target specificity. The microbubble cavitation induced by FUS can act mechanically on the TJs, creating reversible gaps in the endothelial walls [11,12,13,14,15], through which the passive transport of macromolecules to the brain is increased in a controlled and safe manner [16,17,18,19,20,21,22]. Few studies have suggested that BBB opening by FUS may also cause the temporary down-expression of P-gp on vessel walls, the duration of which was dependent on acoustic pressure (AP). P-gp was shown to fully recover three days after FUS application at low AP (e.g., peak negative pressure—PNP of 0.5 MPa, in situ) [23,24] whereas, at a higher AP of 0.8 MPa (similar to levels used in brain tumors trials [25,26]), P-gp does not return to baseline after three days [24]. However, despite these early findings, very little is known about the joint dynamics of the FUS-induced regulation of efflux transporters expressed at the BBB, and more data are needed to compare the dynamics of the expression of P-gp and BCRP over a longer time after FUS. For the first time, this pilot study aims to bridge these gaps by investigating the effects of FUS-mediated BBB-opening on P-gp and BCRP expressions at 0.8 MPa over 14 days.

## 2. Results

**Figure 1:**
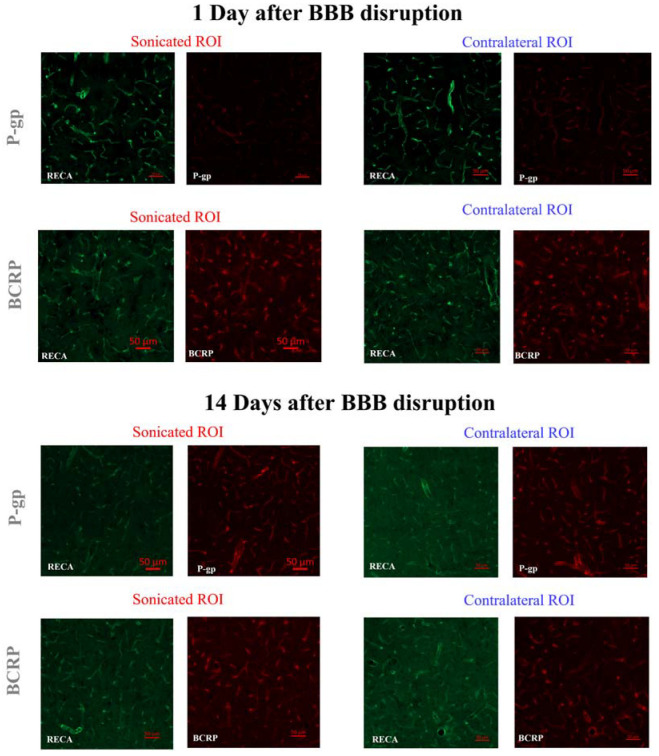
Representative immunohistochemical staining of P-gp and BCRP on sonicated and contralateral brain tissues, obtained on day 1 and 14 days after FUS-induced BBB disruption.

Figure 1 shows the representative P-gp/BCRP stainings of the sonicated and contralateral brain tissues obtained on days one and 14 after FUS-induced BBB disruption. After BBB disruption, P-gp was down-regulated on day one when compared to BCRP and 14 days after sonication. At no time point was BCRP down-regulated by FUS. Further protein stainings that confirm these results are shown in the Supplementary Materials (Figures S1–S4).

**Figure 2:**
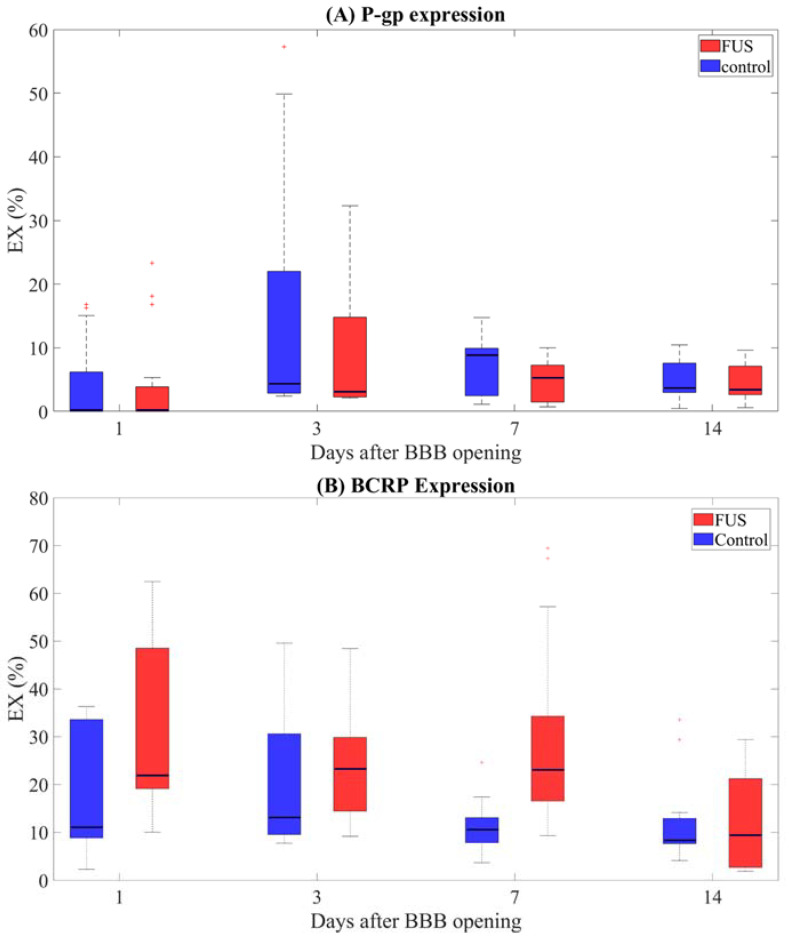
P-gp (**A**) and BCRP expressions (**B**), evaluated over 18 ROIs/time points in both sonicated (in red) and contralateral untreated brain regions (in blue).

The levels of each differentially expressed protein up to 14 days after FUS are shown in Figure 2 for both brain hemispheres (sonicated vs. contralateral). Three animals were used to assess the expression of each protein at each time point. For each animal, the protein expressions were assessed in two slices that were centered in the focal spot region, with three ROIs per animal for each hemisphere, yielding a total of 18 ROIs for each time point.

Although the median P-gp expression in the sonicated regions appears lower than in the contralateral regions, such differences are not statistically significant (the *p*-value associated with the hemisphere effect is 0.92). This result is also confirmed by the *p*-values resulting from the posthoc analysis and is shown in Table 1. These findings suggest that an AP of 0.8 MPa affects P-gp expression throughout the brain with signs of increased effects on the sonicated side.

When comparing P-gp expression in the sonicated or contralateral regions at different time points, it is possible to detect statistically significant changes in the expression of this protein over time (the effects of the day and of the “day × hemisphere” interaction terms were associated with *p*-values of 0.012 and 0.0017, respectively). The *p*-values resulting from all statistical comparisons in the sonicated and contralateral ROIs are shown in Table 2 and Table 3, respectively. Our findings imply that one day after sonication, P-gp expression was lower in both the sonicated and contralateral regions when compared to the following days (*p* < 0.05 for all comparisons between Day 1, Day 3, and Day 7, performed in single hemispheres separately). In addition, P-gp expression did not change from Day 3 to Day 14 in both hemispheres. Regarding BCRP, we found a statistically significant dependence of its expression on the brain hemisphere (*p* = 6.74 × 10^−5^), together with a significant “day × hemisphere” interactive effect (*p* = 3.34 × 10^−5^). No effect of the days since sonication was found (*p* = 0.52). The Mann–Whitney U tests suggest thae following:

(i) Up to seven days after sonication, BCRP is significantly higher in the sonicated area compared with the contralateral brain regions (*p*-values fdr corrected <0.05, Table 1);
(ii) Two weeks after sonication, BCRP expression in the treated and untreated regions was similar (p-value fdr corrected > 0.05 when comparing hemispheres at day 14);
(iii) BCRP expression remains constant in the two hemispheres within a week after FUS (p-values fdr corrected >0.05 from all comparisons between time points, Table 2 and Table 3);
(iv) Interestingly, in the sonicated regions, BCRP appears to be significantly more expressed for the first seven days compared to two weeks postFUS (Table 2). The same effect was not observed on the contralateral side (Table 3).

**Figure 3:**
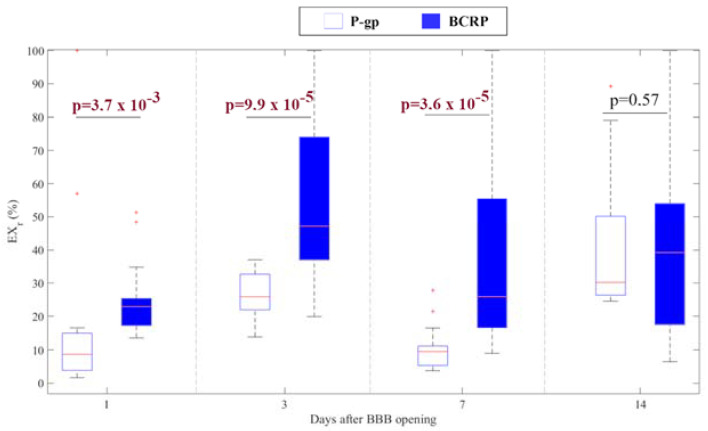
Boxplots of both P-gp and BCRP expressions in the sonicated regions normalized to the expressions in the contralateral ROIs evaluated at different time points within two weeks after FUS application.

Figure 3 shows the percentage of voxels (EXr (%)) expressing P-gp or BCRP in the sonicated tissues compared to the contralateral hemispheres. BCRP expression is significantly higher than P-gp expression within a week after BBB disruption (all *p*-values < 0.05 when comparing their expressions at 1, 3, and 7 days after sonication), while their expressions are similar at two weeks after FUS application (*p*-value = 0.57).

## 3. Discussion and Conclusions

In this pilot study, for the first time, we investigated the impact of FUS-mediated BBB disruption at moderate AP on the expression of both P-gp and BCRP for up to two weeks postsonication. Our results show that at 0.8 MPa, P-gp is down-expressed for up to three days after sonication. In addition, corroborating a previous study [24], this transport is down-regulated in the treated region and the contralateral hemisphere. On the contrary, BCRP was over-expressed in the treated regions only for up to seven days after FUS application and fully recovered within two weeks. Overall, these results suggest that (i) FUS can alter the expression of P-gp but also impacts BCRP; (ii) full recovery is achieved within two weeks.

### 3.1. Implications for Drug Delivery Strategies

Our findings can help shed light on the biological mechanisms induced by FUS and explain why, in some cases, it cannot improve molecular uptake in the brain. Even at optimal postFUS times (i.e., when the P-gp is maximally inhibited), P-gp down-expression may remain insufficient to block its function [27]. In addition, our results show that P-gp inhibition is accompanied by BCRP over-expression that compensates for the P-gp partial loss of action. This suggests that administering inhibitors of the BCRP proteins in conjunction with FUS application may be beneficial for further enhancing molecular deliveries of ABC transporter substrate molecules (such as tyrosine kinase inhibitors) to the brain. In addition, investigating the impact of FUS on brain tumors where this transporter is over-expressed may help offer alternative mechanisms to fight the disease [28,29,30]. For example, it may inspire new drug designs based on delivery strategies in this framework. Indeed, our preliminary results suggest that this method may improve the delivery of the substrates of P-gp that are not substrates of BCRP, which FUS overexpresses.

### 3.2. Study Limitations and Future Directions

This is the first study demonstrating that FUS-induced BBB disruption is accompanied by the dysregulation of both P-gp and BCRP, the main efflux transporters at the BBB, in opposite directions. Future studies should be conducted with a larger animal cohort to increase the statistical power of the results.

Here we set out to understand the acute and very focal effects of FUS application targeted to a single point in the brain (i.e., a protocol typically found in clinical applications [26,31]). The limited brain volume (2 × 2 × 6 mm^3^) exposed to the same acoustic energy did not allow us to assess P-gp and BCRP expression using immunohistochemistry in conjunction with Western blotting. For our purposes, the study design also allowed a cross-comparison of our results with a previous study, showing the effects of n of the same acoustic intensity on P-gp expression [24].

Future studies, investigating the effects of FUS using a much larger sonication volume (e.g., whole thalamus [23]) will allow protein expressions to be assessed by immunohistochemistry along with more quantitative methods (e.g., Western blot).

In addition, since the permeabilization of the BBB under FUS strongly depends on the acoustic parameters used during sonication (e.g., AP, FUS frequency, and duty cycle) as well as the type of MBs, further studies are needed to investigate the functional impact of these parameters on the expression of both of the efflux transporters. The generalization of the present findings (obtained using rodents) to other mammalian brains, including human beings, also remains an open question since it is known that ABC transporter expressions vary among species.

## 4. Materials and Methods

### 4.1. Animals

All experiments were performed in accordance with the recommendations of the European Community (86/609/EEC) and the French legislation (decree no. 87/848) for the use and care of laboratory animals. All animal experiments were approved by the Comité d’Éthique en Expérimentation Animale du Commissariat à l’Énergie Atomique et aux énergies alternatives Direction des Sciences du Vivant Ile de France (CETEA CEA DSV IdF) under protocol ID_ 12-058. Experiments were performed on 12 Fischer male rats (~350 g). Animals were anesthetized by using a mixture of air, oxygen, and 1.5% isoflurane, and the heads were shaved before being placed in the MRgFUS device. A heater was used to keep the animals close to their physiologic temperature of 36 ± 1 °C. Body temperature and respiration rate were continuously monitored during the experiments. A catheter was inserted in the caudal vein to inject both microbubbles and MRI contrast agent (CA) from outside the scanner.

### 4.2. Experimental Protocol

**Figure 4:**
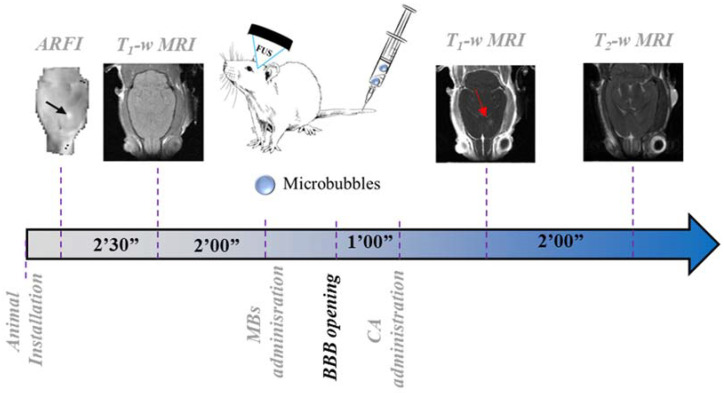
Experimental protocol for in vivo experiments and timelines for the BBB permeabilization procedure and MRI acquisition. After the installation of the animal in the MRI scanner, the position of the FUS focal spot was confirmed with an MR-ARFI sequence. This acquisition was then followed by a T_1_-w scan acquired before BBB opening. One minute after FUS application and MRI-CA administration, T1-w (2 min acquisition) and T2-w (2 min) images were acquired to evaluate the extension of the BBB disruption and the presence of damages, respectively.

The experimental protocol is depicted in Figure 4, along with timelines for the BBB permeabilization procedure and MRI acquisition. After the installation of the animals in the MRI scanner (7 T/90 mm bore hole Pharmascan scanner, Bruker, Ettlingen, Germany), Acoustic Radiation Force Imaging (ARFI) [32] was acquired to confirm FUS targeting in the brain (MSME, TE/TR = 28/1080 ms, matrix = 64 × 64 × 5, res = 0.5 × 0.5 × 2 mm^3^, total duration: 2′30′′). T_1_-weighted images where then acquired (T_1_-w, MSME sequence, TE/TR = 8.3/300 ms, matrix dimension = 256 × 256 × 10, resolution= 0.125 × 0.125 × 1 mm^3^, 3 averages, acquisitions time = 2 min) before FUS application. The acoustic treatment consisted of 3 ms bursts, every 100 ms, over a period of one minute, with an estimated focal PNP in the brain of 0.8 MPa, taking into account skull transmission factor based on [33]. An AP of 0.8 MPa was chosen since it is a typical level used to permeabilize the BBB in brain tumors in preclinical and clinical trials [25,26]. The treatment was performed with an MR-compatible FUS transducer (1.5 MHz central frequency, diameter: 25 mm, focal depth: 20 mm, Imasonic, Voray-sur-l′Ognon, France) connected to a therapeutic programmable FUS generator (Image-Guided Therapy, Pessac, France).

FUS was applied at a single point in the striatum (focal spot- FS: 2 × 2 × 6 mm^3^) [34]. A 200 µL bolus of SonoVue microbubbles (Bracco, Italy) was injected via a tail-vein catheter approximately 5 s before sonication. Approximately 30 s after FUS application, a Gadolinium-based CA (Dotarem^®^, 1 nm diameter, 1.6 mL/kg) was injected via a catheter. Approximately 30 s after the CA-administration, T_1_-w acquisition started. At the end of each experimental session, T_2_-weighted (T2-w) images were acquired through a RARE sequence (TE/TR = 10/3800 ms, RARE factor = 8, matrix = 128 × 128 × 32, res = 0.225 × 0.225 × 0.5 mm^3^) to verify the absence of any hemorrhage or edema due to the BBB permeabilization protocol. 

### 4.3. Immunohistochemistry

BCRP and P-gp expressions were evaluated through histology performed on three rats per time point at 1, 3, 7, and 14 days after BBB opening. The extracted brains were sliced using a microtome (slices thickness = 30 µm). The endothelial cells were stained by RECA-1 antibody (mouse anti-RECA-1 from Abcam, ab9774, diluted at 1/2000), while P-gp and BCRP expressions were evaluated through rabbit anti-P-gp (Abcam ab170904, diluted at 1/100) and rabbit anti-BCRP from Abcam ab207732 (diluted at 1/100); all used after antigen retrieval process (acetic acid 33%/ethanol pure 66%; 10 min −30 °C) and the block of permeabilization process (Donkey serum 5% +BSA 1% + triton 1%). Representative negative controls of the immuno-stained regions are shown in Figure S5. For each double staining P-gp/RECA-1 and BCRP/RECA-1, the brain slides were processed through the following protocol:

(i) Wash 2 × 5 min with PBS (concentration of 0.01 M);
(ii) Application of antigen retrieval process (ac/eth) followed by blocking/permeabilization protocols (2 h);
(iii) Wash with PBS;
(iv) Incubation of the first antibody (1 h for P-gp/2 h for BCRP);
(v) Wash 2 × 5 min with PBS;
(vi) Incubation of the RECA-1 antibody for 1 h;
(vii) Wash 2 × 5 min with PBS;
(viii) Incubation for 1 h of secondary Dk anti-Rabbit Alexa647 and the secondary Dk anti-mouse Dylight488;
(ix) Wash 3 × 5 min with PBS;
(x) Application of ProLong with Dapi (mounting media, from Thermo Fisher, Waltham, MA, USA, ref P36931).

Histological images were obtained using an AxioObserver Z1 microscope (Zeiss, Jena, Germany) with an MRm camera and analyzed using Carl Zeiss AxioVision software. Mozaic scans cover the fields of view through the Tile function of the software.

### 4.4. Histological Findings Evaluation

**Figure 5:**
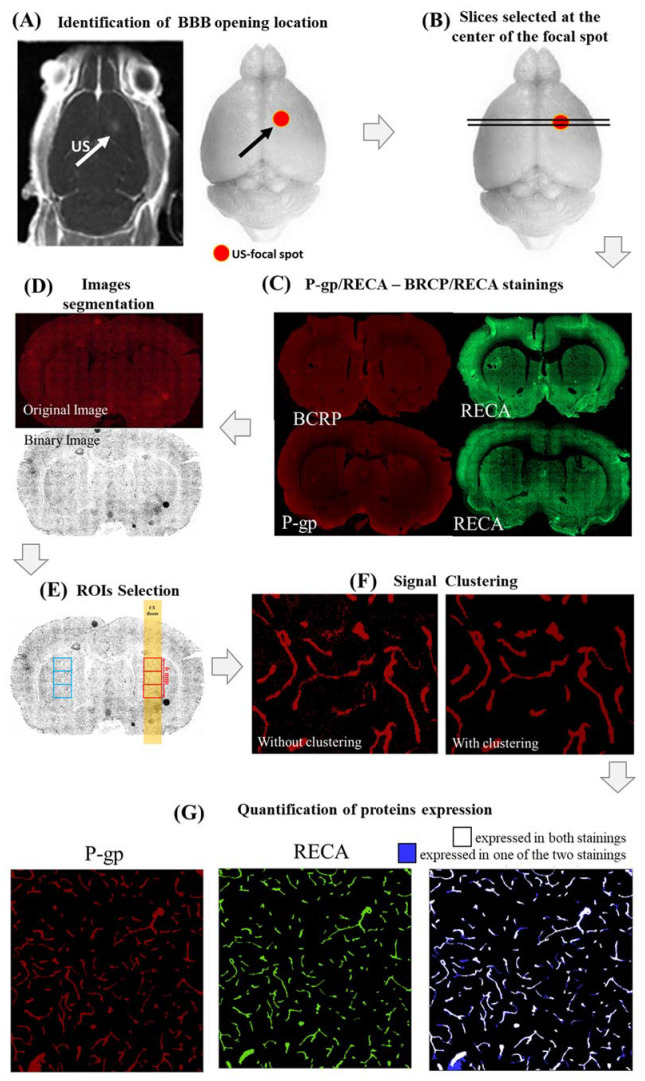
Pipeline used to evaluate P-gp, BCRP, and RECA expressions in rat brains after FUS. In all cases, the center of the focal spot was identified in the postcontrast T_1_-w images acquired right after BBB disruption and MRI-CA administration (**A**). (**B**): four slices centered in the focal regions were selected (all of them were stained with RECA, while groups of two slices were stained with P-gp and RECA, respectively). Figure (**C**) shows two representative slices (one per protein) with the respective RECA stainings. All slices were binarized (**D**), then three squared regions (2 mm wide) were selected at the center of the focal spot, both in the sonicated and contralateral hemispheres (**E**). Expressions in all ROIs were clustered to remove voxels belonging to the background (**F**). As the last step, the expression of each protein was evaluated as the percentage of voxels expressing both RECA-1 and BCRP or P-gp (blue voxels in Figure (**G**)) [Microscope images at 10× magnification].

Figure 5 shows the pipeline used to evaluate the histological findings quantitatively:

(i) First, the center of the BBB opening was identified in all rat brains by looking at T_1_-w images, acquired immediately after FUS application and CA injection (Figure 5A);
(ii) Four brain slices positioned around the center of the FUS focal spot were stained for both RECA-1/P-gp (2 slices) and RECA-1/BCRP (2 slices), as shown in Figure 5B,C;
(iii) A custom MATLAB script (MathWorks, USA) thresholded and binarized the histological images for all stainings, as follows (Figure 5D):
  - Distributions of P-gp/BCRP and RECA expressions were first fitted in the red and green color channels, respectively, through Gaussian functions. The mean (M) and the standard deviation (σ) of each of these distributions were calculated;
  - Binarized images were obtained by excluding hyper/hypointense meaningless background voxels (i.e., with values lower than [M − 2.5σ] or higher than [M + 2.5σ]);
(iv) For each slice and staining, three adjacent square regions of interest (ROIs) of 1000 voxels per side (about 2 mm) centered on the focal spot and the contra-lateral side were defined (Figure 5E). The ROIs were chosen to be large enough to cover the ultrasound spot dimensions (2 mm wide, 6 mm long). Finally, this led us to 6 analyzed ROIs per staining and per rat (18 ROIs in total per single hemisphere/time point). Our sample size was similar to that used in [24] so that we had sufficient statistical power to assess the effects of FUS on the expression of both proteins;
(v) In order to remove the remaining spurious noisy voxels, the voxels in each of these ROIs were clustered using a custom MATLAB code (Figure 5F). The value of the suprathreshold voxel among eight neighbors was assigned to one cluster. This technique was used for all BCRP, P-gp, and RECA-1 stainings;
(vi) The expressions of P-gp and BCRP were defined as the percentage of voxels in each ROI expressing both proteins (P-gp or BCRP) and RECA-1. These voxels are depicted in blue in Figure 5G for a representative ROI in which both P-gp and RECA-1 were expressed.

To assess the effects of FUS on the expressions of BCRP and P-gp at different time points, the percentage of voxels expressing the two proteins was compared to the contralateral hemisphere: ($EX_{r}\left(\%\right)=\frac{EX_{u}}{EX_{c}}\cdot 100$).

### 4.5. Statistical Analysis

Both protein expressions were independently compared between the sonicated and untreated contralateral brain hemispheres at 1, 3, 7, and 14 days after FUS. We evaluated the hypothesis of a day-specific effect on the difference between contralateral vs. sonicated hemispheres (i.e., a “day × hemisphere” interaction) on both proteins’ expression. To test this hypothesis, we employed generalized linear mixed models with diagonal covariance structures, which included both “the number of days elapsed since the application of FUS” and the “hemisphere” (contralateral vs. sonicated) as fixed effects, together with a “day × hemisphere” interaction term. All models included the animal as a random effect.

EX_r_ values related to P-gp and BCRP expressions were compared between the time points through generalized linear mixed models with diagonal covariance structure, which included day after treatment as a fixed effect and animal as a random effect.

Posthoc pairwise comparisons were conducted using Mann–Whitney U tests. The resulting p-values were corrected for with multiple comparisons (false discovery rate—fdr, alpha = 0.05) across the number of tests. The use of this test was justified by the nonhomogeneity of the variances in the data (see *p*-values resulting from the Barletee tests performed when comparing the protein expressions across time points, shown in Table S1). For all statistical tests, the differences in protein expression were considered statistically significant when associated with *p*-values of less than 0.05.

## Figures and Tables

**Table 1:** The *p*-values resulting from the Mann–Whitney tests performed to compare P-gp and BCRP expression between the sonicated and contralateral brain regions at 1, 3, 7, and 14 days after FUS application (18 ROIs per brain hemisphere, at each time point). The *p*-values were corrected for multiple comparisons (fdr, alpha = 0.05). Corrected p-values less than 0.05 are flagged with one star (*).

P-gp Expression		BCRP Expression	
Days After Sonication	*p*-Value	Days After Sonication	*p*-Value
Day 1	0.88	Day 1	0.028 *
Day 3	0.46	Day 3	0.5
Day 7	0.22	Day 7	2.52 × 10^−4^ *
Day 14	0.93	Day 14	0.93

**Table 2:** *p*-values from P-gp and BCRP expression comparisons in 18 sonicated ROIs at all time points. *p*-values were corrected for multiple comparisons (fdr, alpha = 0.05). Corrected p-values less than 0.05 are flagged with one star (*).

P-gp Expression		BCRP Expression	
Comparison	*p*-Value	Comparison	*p*-Value
Day 1/Day 3	6.8 × 10^−3^ *	Day 1/Day 3	0.51
Day 1/Day 7	6.8 × 10^−3^ *	Day 1/Day 7	1.00
Day 1/Day 14	0.02 *	Day 1/Day 14	3.0 × 10^−3^ *
Day 3/Day 7	0.51	Day 3/Day 7	0.50
Day 3/Day 14	0.51	Day 3/Day 14	1.36 × 10^−2^ *
Day 7/Day 14	0.93	Day 7/Day 14	3.00 × 10^−3^ *

**Table 3:** *p*-values from P-gp and BCRP expression comparisons in 18 contralateral ROIs at all time points. *p*-values were corrected for multiple comparisons (fdr, alpha = 0.05). Corrected p-values less than 0.05 are flagged with one star (*).

P-gp Expression		BCRP Expression	
Comparison	*p*-Value	Comparison	*p*-Value
Day 1/Day 3	4.80 × 10^−3^ *	Day 1/Day 3	0.74
Day 1/Day 7	1.10 × 10^−2^ *	Day 1/Day 7	0.32
Day 1/Day 14	0.064	Day 1/Day 14	0.26
Day 3/Day 7	0.48	Day 3/Day 7	0.104
Day 3/Day 14	0.51	Day 3/Day 14	0.064
Day 7/Day 14	0.36	Day 7/Day 14	0.69

## Data Availability

The data presented in this study are available on request from the corresponding author.

## Supplementary Materials

The following supporting information can be downloaded at: https://www.mdpi.com/article/10.3390/ijms232415488/s1.
