# Supplementary material for: Regulation of P-glycoprotein and Breast Cancer Resistance Protein Expression Induced by Focused Ultrasound-Mediated Blood-Brain Barrier Disruption: A Pilot Study
Source: Int J Mol Sci. 2022 Dec 7;23(24):15488. doi: 10.3390/ijms232415488 (PMC9779754; doi:10.3390/ijms232415488)
Supplement: Supplementary file 1 [file ijms-23-15488-s001.zip › ijms-1964494-supplementary.pdf]

Supplementary material

Regulation of P-glycoprotein and breast cancer resistance protein expression induced by focused ultrasound-mediated blood-brain barrier disruption: a pilot study

Allegra Conti, Francoise Geffroy, Hermes A. S. Kamimura, Anthony Novell, Nicolas Tournier, Sébastien Mériaux and Benoit Larrat

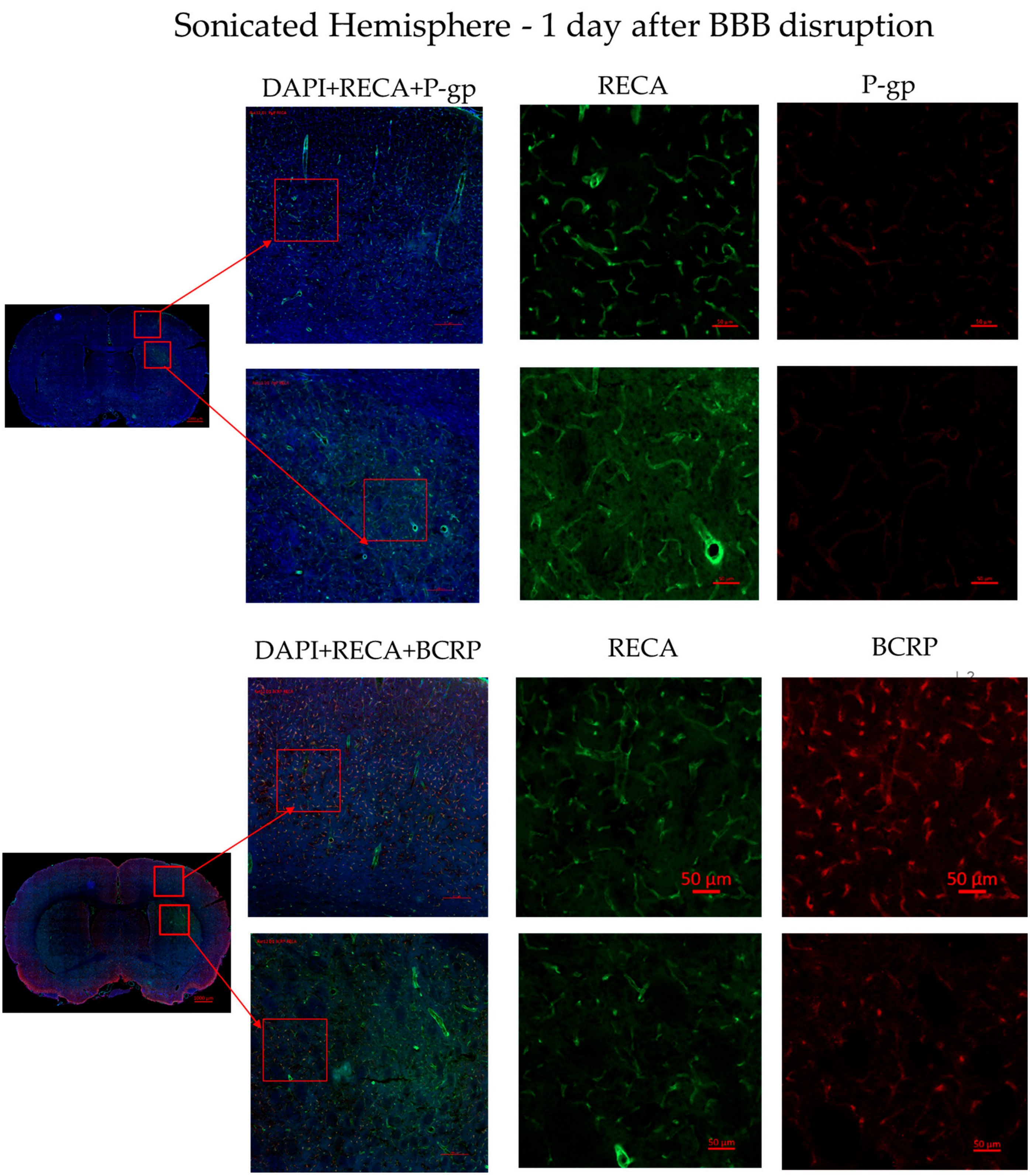

Figure S1: exemplar P-gp and BCRP expressions in sonicated hemisphere at one day after FUS application.

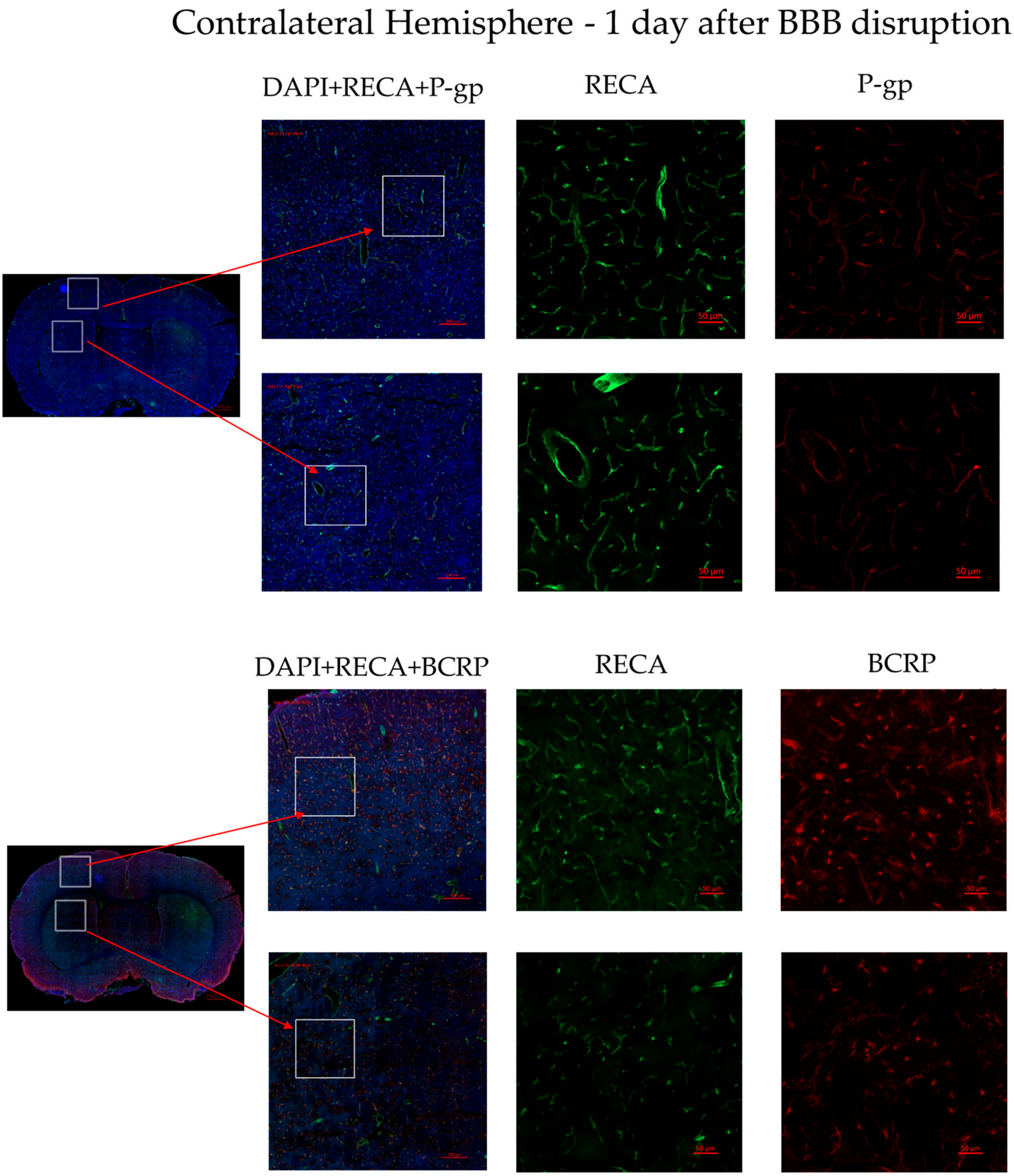

Figure S2: exemplar P-gp and BCRP expressions in contralateral hemispheres at one day after FUS application.

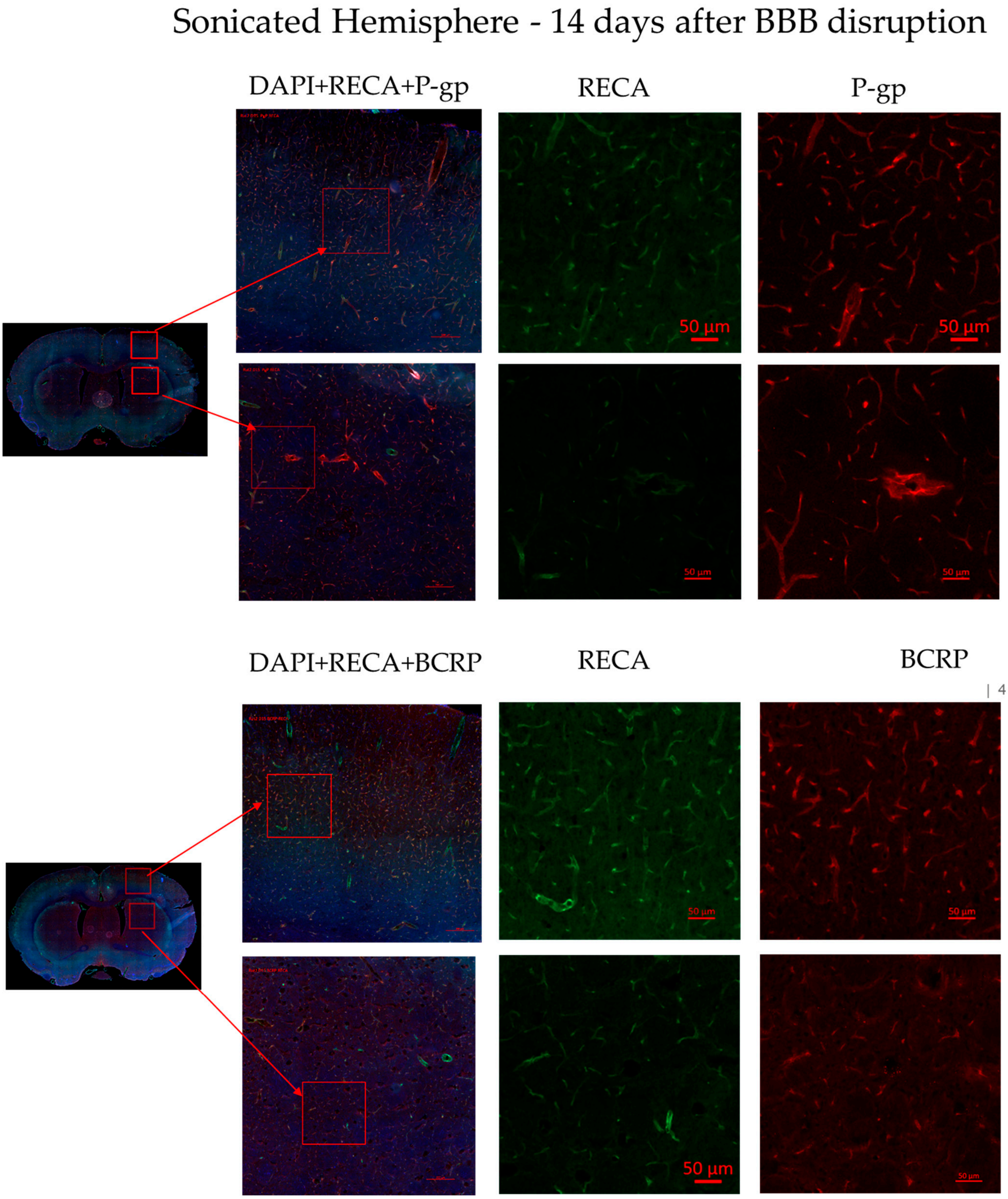

Figure S3: exemplar P-gp and BCRP expressions in sonicated hemispheres 14 days after FUS application.

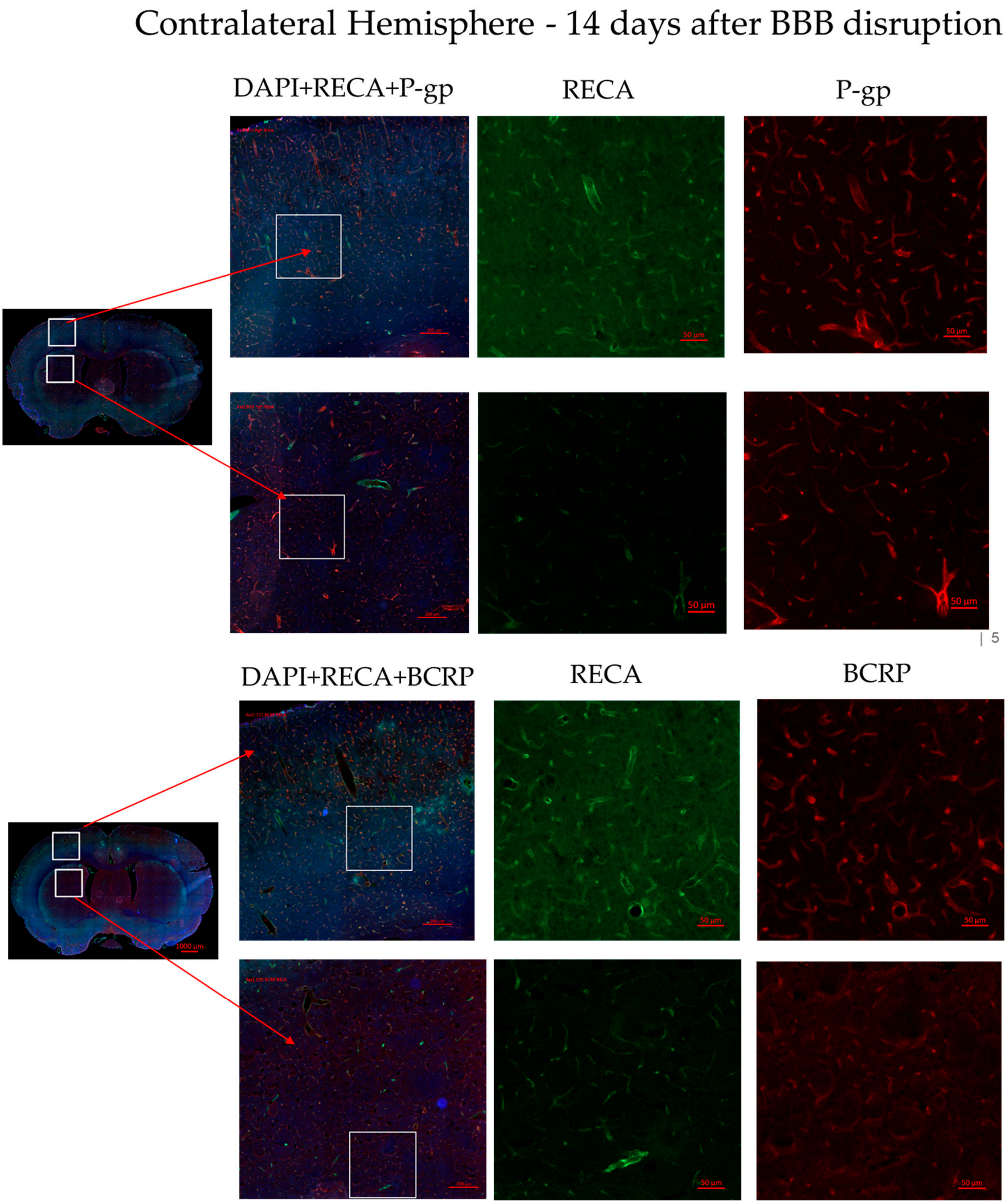

Figure S4: exemplar P-gp and BCRP expressions in sonicated hemispheres 14 days after FUS application.

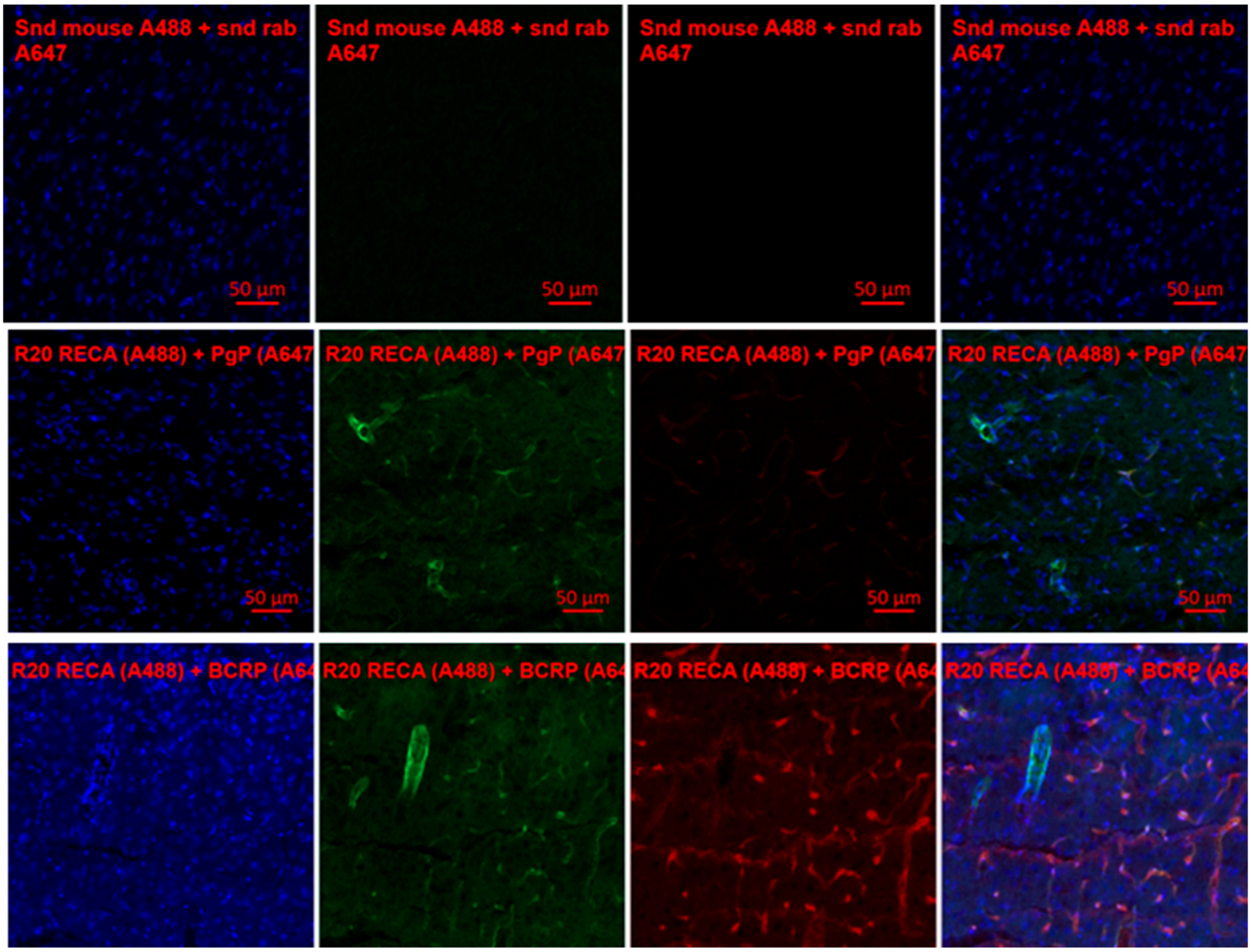

Figure S5: Exemplar negative controls of immuno-stained regions obtained in sonicated regions, 24 hours after FUS application. First raw: secondary antibodies. Second raw: P-gp and RECA stainings. Third row: BCRP and RECA stainings.

Table S1. p-values resulting from Bartlett tests performed to evaluate variance homogeneities of protein expressions across time points.

**Protein [Hemisphere]**	**p-value**
P-gp [sonicated hemisphere]	2.3e-8
P-gp [contralateral hemisphere]	6.7e-18
BCRP [sonicated hemisphere]	2.9e-3
BCRP [contralateral hemisphere]	5.1e-4
